# Potential of *Penicillium* Species in the Bioremediation Field

**DOI:** 10.3390/ijerph6041393

**Published:** 2009-04-09

**Authors:** Ana Lúcia Leitão

**Affiliations:** 1 Grupo de Ecologia da Hidrosfera, Faculdade de Ciências e Tecnologia, Universidade Nova de Lisboa, Quinta da torre, 2829-516 Caparica, Portugal; 2 Unidade de Biotecnologia Ambiental, Quinta da Torre, 2829-516 Caparica, Portugal

**Keywords:** Penicillium, biodegradation, bioremediation

## Abstract

The effects on the environment of pollution, particularly that caused by various industrial activities, have been responsible for the accelerated fluxes of organic and inorganic matter in the ecosphere. Xenobiotics such as phenol, phenolic compounds, polycyclic aromatic hydrocarbons (PAHs), and heavy metals, even at low concentrations, can be toxic to humans and other forms of life. Many of the remediation technologies currently being used for contaminated soil and water involve not only physical and chemical treatment, but also biological processes, where microbial activity is the responsible for pollutant removal and/or recovery. Fungi are present in aquatic sediments, terrestrial habitats and water surfaces and play a significant part in natural remediation of metal and aromatic compounds. Fungi also have advantages over bacteria since fungal hyphae can penetrate contaminated soil, reaching not only heavy metals but also xenobiotic compounds. Despite of the abundance of such fungi in wastes, penicillia in particular have received little attention in bioremediation and biodegradation studies. Additionally, several studies conducted with different strains of imperfecti fungi, *Penicillium* spp. have demonstrated their ability to degrade different xenobiotic compounds with low co-substrate requirements, and could be potentially interesting for the development of economically feasible processes for pollutant transformation.

## 1. Introduction

As a result of adaptation to their environment, fungi have developed unique bioremediation properties. The objective of this review is not to be exhaustive, but rather to summarize the remediation potential of *Penicillium* strains and the environmental implications, focusing on recent developments in this area.

Fungi have been harnessed by humans for several applications for thousands of years. They are well known to degrade, or cause deterioration to, a wide variety of materials and compounds, processes known as mycodegradation and mycodeterioration, respectively [1]. Polyethylene degradation is a good example of mycodegradation. *Penicillium simplicissimum* YK is able to degrade polyethylene, with a molecular weight of 400 to 28,000 [2].

*Penicillium* belongs to the phylum *Ascomycota*, however its taxonomic characterization is still a matter of discussion [3] and the difficulties in identifying most *Penicillium* species requires multidisciplinary approaches. Clarification of species concepts in the genus *Penicillium* was supported mainly by morphological characteristics. Raper and Thom, for example, based *Penicillium* taxonomy classification on the combination of macroscopical (such as colony texture and color) with micromorphological features [4]. In spite of the fact this feature was regarded as subjective by Pitt and Stolk and Samson, the color of the conidial mass has been pointed out to be a species-specific characteristic that varies in concomitance with distinctive morphological features [5–7]. Dorge *et al.* proposed a method for direct identification of pure *Penicillium* species using image analysis [8].

The various species of *Penicillium* can colonise many different environments. They are common in soils, in foods, in drinks and in indoor air [9]. There are several reasons why *Penicillium* remediation of heavy metal and xenobiotics is important to researchers and practitioners. Fungi are usually slow growing and often require substrates for cometabolism. Therefore, in general, fungi are less efficiently than bacteria in xenobiotic degradation. On the other hand, fungi are more versatile and more suitable in what concerns to the breadth of substrates they can use. Unlike fungi, bacteria are unable to degrade efficiently polycyclic aromatic hydrocarbons (PAHs) with more than four aromatic rings. The morphology and growth characteristics of fungi are responsible for the rapid colonization of substrates. Structural heterogeneity is considered to be one of the most important characteristics of fungal pellets. This feature depends on density of packing and affects limitation of both nutrient and oxygen. Fungi can form or not pellets, depending on several factors such as medium composition, inoculum concentration and preparation, agitation intensity and others [10]. Tightly compact pellets are deficient in nutrient and oxygen and generally are hollow in the center, due to autolysis of the mycelium. In spite of that pellets with a diameter of less than 400 μm that contains all active cells were observed in *Penicillium chrysogenum* [11].

The ability of most fungi to produce extracellular enzymes for the assimilation of complex carbohydrates without prior hydrolysis makes possible the degradation of a wide range of pollutants. They also have the advantage of being relatively easy to grow in fermenters, thus being suited for large scale production. Another advantage is the easy separation of fungal biomass by filtration due to its filamentous structure. In comparison to yeasts, filamentous fungi are less sensitive to variations in nutrients, aeration, pH, temperature and have a lower nucleic content in the biomass. In addition, several *Penicillium* strains have been shown to be able to live in saline environments, an advantage of these microorganisms over the others in the bioremediation field. *Penicillium* strains generally are halotolerant microorganisms, able to grow either in the presence or in the absence of salt; those halotolerants that are able to grow, above approximately 15% (w/v) NaCl are considered extremely halotolerant. Hypersaline wastes are generated in several industrial activities, such as chemical manufacture, oil and gas production and waste minimization practices. This wastes, commonly designated as produce waters, are constituted by water containing high concentration of salts, oil, organic acids, heavy metals, and radionuclides [12], therefore, the ability of halotolerants to remediate pollutants in the presence of salt is useful for biological treatment without damage to the physically sensitive ecosystem.

Fungal treatment of wastes in Nature has been known for centuries [13]. The ubiquitous presence of fungi has allowed acclimation to some if not most types of wastes. During the last decade, fungi have been used in the treatment of a wide variety of wastes, wastewaters, and the role of fungi in the bioremediation of various hazardous and toxic compounds in soils and sediments has been established. Fungi have also demonstrated the ability to remove heavy metals and to degrade, in some cases mineralize, phenols, halogenated phenolic compounds, petroleum hydrocarbons, polycyclic aromatic compounds, and polychlorinated biphenyls [1].

## 2. Heavy Metals

Heavy metals contamination, which has increased sharply over the last century due to increasing industrialization, imposes stress on organisms and their presence poses environmental-disposal problems due to their non-degradable and persistent nature. Therefore, the introduction of cleaner technologies is not only socially responsible, but has also been shown to lead to increased productivity, competitiveness and profitability. Heavy metals, such as cadmium, lead and mercury, are known for their high toxicity and impact as they have been connected to major health hazards. These could be followed by arsenic and chromium as they were used industrially in large quantities for a long time. Arsenic has been reported as “the largest poisoning in the history of mankind”, and millions of people in Bangladesh and India drink and irrigate cultures with contaminated water. Indeed, arsenic contamination problems come not only form anthropogenic sources, as it was recently reported that natural leaching from local geological formations can cause also toxic effects when waters used. Similarly for mercury, both anthropogenic (gold mining in Brazil) and natural (sub-arctic lakes) sources are known as the cause of health problems in local populations. Nowadays the scientific attention is mainly focused on four sources of heavy metals, as a consequence of their environmental impact [14]:

1. acid mine drainage (AMD), associated with mining operations;
2. electroplating industry waste solutions;
3. coal-based power generation (high coal quantities);
4. nuclear power generation (uranium mining and waste generation).

Speciation of heavy metals is important, because different oxidation states of an element have different toxicity. This fact implies that the development of methods that allow selective removal of different elements is a complex task. It is known that most metals occur in cationic forms, but the most toxic forms of arsenic and chromium, for example, are anionic species (arsenite and chromate, respectively). Arsenite is 10 times more toxic than arsenate and 70 times more toxic than the methylated species [14,15].

Elimination of heavy metals from industrial wastewater is commonly done by means of chemical precipitation, ion exchange, solvent extraction, electrochemical treatment, reverse osmosis, membrane technologies, evaporation recovery and chemical oxidation-reduction which are complex and expensive processes that frequently result in the production of toxic products and thus formation of secondary sources of environmental pollution. Another major drawback is the lack of ability for removal of heavy metals in low concentrations, especially when the concentrations are in the 1–100 mg/L range [16]. Therefore, removal of toxic heavy metals to an environmentally safe level in a cost effective and environment friendly manner assumes great importance. Microorganisms have been used as such a low cost method to remove metals from effluents [14], with fungi known to be more tolerant to metals and to have a higher microorganism surface to volume ratio than bacteria or actinomycetes [17]. Fungi are not only a major component of the biota in soils and mineral substrates, but also under certain environmental conditions (low pH), they can be efficient biogeochemical agents and bioaccumulators of soluble and particulate forms of metals. Among them, *Penicillium* spp. are described as prominent ones [18].

The process of biosorption and bioaccumulation of metals by fungi is not new. These microorganisms are known to detoxify metals by several mechanisms including ion exchange, chelation, adsorption, crystallization, valence transformation, extra and intracellular precipitation and active uptake [19,20]. In other words the accumulation of metals from solutions by fungi can be divided into three categories: (1) biosorption of metal ions on the surface of fungi, (2) intracellular uptake of metal ions, and (3) chemical transformation of metal ions by fungi [1]. Biosorption can use both living and non-living biomass in the processes as it frequently exhibits marked tolerance towards adverse conditions like heavy metals. In the case of biosorption processes for heavy metal concentration using non-living biomass, the metabolic activity needed for intracellular metal accumulation is absent, meaning that nonliving fungal biomass does not depend on growth, metabolic energy, and transport needs. The problem of toxicity of metals does not affect this type of biomass, which is seen as one major advantages of biosorption. Living fungal biomass is required in the last two categories. This process is a much more complex bioaccumulation phenomenon based on active metabolic transport, and biosorption is based mainly on the “affinity” between the (bio-)sorbent and sorbate [14]. The biosorption phenomenon is a direct competitor of ion exchange in what concerns to wastewater treatment systems application for remedial processes. Therefore, the importance of the field of biosorption cannot be overestimated. Within this framework, not only can more effective engineering process design/optimization tools be developed, but also a contribution from the marketing and biomass supply sides would be most useful and very desirable for the start-up of viable commercial enterprises [14].

Metal sorption and accumulation depends on diverse factors, such as pH, temperature, organic matter, ionic speciation and the presence of other ions in solutions, which may be in competition, etc. [21,22].

In recent years, the biosorption process using organism biomass has been studied extensively as an important preconcentration-separation method for heavy metals at trace level [23]. Therefore, biosorption has been defined as the property of certain biomolecules (types of polymer or biomass) to bind and concentrate selected ions or molecules from aqueous solutions.

Fungi under stress develop several mechanisms in order to tolerate adverse conditions. They develop adaptation through a temporary alteration in their developmental patterns or by modifications on physiological characteristics, depending on the toxicity of the metals, which in turn is influenced by the concentration and by the salt form in which the metal exists [24].

Recently, four halotolerant *Penicillium* were isolated from mangroves and salterns, selected on the basis of the variations in morphology of the penicillial heads and by their resistance to lead, copper and cadmium salts [24]. It was reported that all four isolates could resist Pb(II) at a concentration of 7.5 mM, with no decrease in growth up to 5 mM and with minimal changes in growth pattern and morphology compared to that of Cu(II) and Cd(II). Three of the strains were resistant at least to 2 mM of Cu(II). Cadmium response stresses were different dependent on strain and salt assayed. The cultures grown in presence of metals showed striking variations in colony appearance, morphology, sporulation and in pigment production as compared to the controls; these changes were more pronounced with increasing metal concentrations [24].

A *Penicillium* sp. isolated from heavily polluted streams near an industrial area in La Plata (Argentina) was able to grow and remove a 100-fold higher Cd level after 13 days of incubation by an absorption process [25].

**Figure 1.**
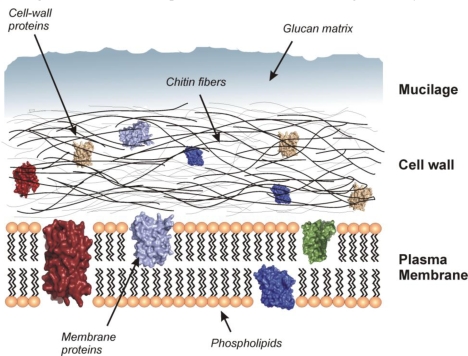
Schematic representation of the outer fungal cell layers.

Fungal cell walls can act as a cation exchanger due to their negative charge originating from the presence of different functional groups, e.g. carboxylic, phosphate, amine or sulfhydryl, in different wall components (hemicelluloses, pectin, lignin, etc.) [17]. The relative importance of each functional group is often difficult to resolve [26]. Cell walls of fungi are rich in polysaccharides and glycoproteins such as glucans, chitin, mannans and phospho-mannans (Figure 1). These polymers provide abundant sources of metal binding ligands. The cell wall structures of fungi present a multi-laminate architecture where up to 90% of their dry mass consists of amino or non-amino polysaccharides [27]. In general, the fungal cell wall can be considered as a two-phase system consisting of a chitin skeleton framework embedded in an amorphous polysaccharide matrix [27].

*Penicillium digitatum* mycelium can accumulate uranium from aqueous solutions of uranyl chloride. Pretreatment of fungal biomass in boiling water or with alcohols, dimethyl sulfoxide, or potassium hydroxide increased the uptake capability to about 10,000 parts per million (dry weight). Meanwhile, formaldehyde killing does not enhance the uranium uptake. Chitin, cellulose and cellulose derivatives from wall fungal interfered in metal ion uptake process. These biopolymers were active in removal of U(VI) [28].

*Penicillium janthinellum* F-13 is able to reduce aluminum toxicity and to produce citric acid, but internal or external sequestration of aluminum seems not to be involved in its tolerance to the high aluminum concentrations [29]. In *Penicillium simplicissimum,* adsorption of Zn(II) is accompanied by the production of citric acid [30].

Recently, the high potential of *P. simplicissimum* to remove Cd(II), Zn(II) and Pb(II) from aqueous solutions was reported [31]. The initial pH significantly influenced Cd(II), Zn(II) and Pb(II) uptake. The sorption capacities of metal ions increased as temperature increased, but decreased with increased in biomass dose. The maximum loading capacity was higher for Pb(II) followed by Zn(II). In binary and ternary metal mixtures, the biosorption capacity of the biomass decreased for each metal ion [31].

The uptake and selective binding of Ni(II), Zn(II), Cd(II) and Pb(II) by the mycelium of *P. digitatum* was demonstrated to be highly pH-sensitive and inhibited below pH 3, whereas uptake of Cu(II) is virtually pH-insensitive. In the case of Ni(II), Zn(II) and Cd(II), acidic solutions inhibit the uptake competitively. The effect on metal uptake of heat killed mycelium (mycelial mass preheated at 100 °C for five min.) was comparable or superior to living preparations, except for Pb(II). Other activators include alkali and dimethyl sulfoxide pretreatment, while formaldehyde inhibited metal uptake. If the equilibrium load for iron was 100, then those for Ni(II), Cu(II) and Zn(II) were 85 to 96, Cd(II) 37 and Pb(II) 33 [32]. *Penicillium digitatum* was described as if it was a mixture of neutral and acidic glycans with no real evidence for cationic amino-functional sites [32].

The immobilization of *Penicillium italicum* on Sephabeads SP 70 in a glass column was reported as a useful biosorption method for the determination of Cd(II), Co(II), Cu(II), Fe(III), Pb(II), Mn(II), and Ni(II). This new matrix could be used at least 100 times without any loss its adsorption properties. The biosorption procedure was described to be easy, safe, rapid, and inexpensive for the preconcentration and separation of trace metals in aqueous solutions [33]. Some examples of the level of heavy metal reduction achievable by biosorption processes are given in Table 1.

The fungus *Penicillium canescence* was described to be able of remove the Cd, Pb, Hg and As ions from aqueous solutions by biosorption [34].The binding of heavy metal ions to *P. canescence* was pH-dependent. The same affinity order was observed under non-competitive and competitive absorption conditions. *P. canescence* showed preference to binding Pb over Cd, Hg and As ions [34].

The ability of *Penicillium purpurogenum* to bind high amounts of Cr(VI) was firstly reported by Say *et al.* [35]. Cr(VI) adsorption capacity increases with time during the first four hours and then levels off toward the equilibrium adsorption capacity. The adsorption of Cr(VI) was clearly pH dependent and loading capacity increased with increasing the pH. The fungal biomass could be reused six times with negligible decrease in biosorption capacities [35].

The use of *Penicillium chrysogenum* to remove metal ions with high efficiency was first reported by Niu *et al.* [18]. At pH 4.5, nonliving *P. chrysogenum* biomass not only removed Pb ions (116 mg/g dry biomass) from aqueous solutions, but also exhibited selectivity for Pb(II) over the other metal ions studied [Cd(II), Cu(II), Zn(II) and As(III)]. The sorption uptake of Pb(II) remained unchanged in the presence of Cu(II) and As(III), it decreased in the presence of Zn(II), and increased in the presence of Cd(II) [18]. Tan and Chen [42] reported that adsorption of metal ions was strongly pH dependent. Alkaline pretreatment increased the adsorption capabilities of *P. chrysogenum*. This effect was more significant for Zn(II) an 3.6-fold increase in adsorption abilities was observed after alkaline pretreatment. In the case of Cr(III) and Ni(II) the adsorption capacity of *P. chrysogenum* mycelium increased only about 45% with respect to the adsorbent system without pretreatment. The adsorbent could be reused at least three times without loss of activity. *P. chrysogenum* mycelium was proposed for large scale Cr(III) elimination from tannery wastewater. In spite of that, the low mechanic intensity, limited reuse and low capacity (10–30 mg/g), seems to create difficulties in order to implement the use of *P. chrysogenum* mycelium to remove metal ions [42]. Tan and coworkers reported a different system for adsorption of Ni(II) on the surface of a molecularly imprinted adsorbent from *P. chrysogenum* mycelium [43]. They observed an adsorption capacity of 40–45 mg/g for Ni(II) on the surface of molecular imprinted adsorbent on *P. chrysogenum* mycelium, two times that of the mycelium adsorbent. The surface imprinted adsorbent had good stability and could be reused 15 times without losing its uptake [43]. A *Penicillium chrysogenum* PTCC 5037 that is formed on cheese and grown very easily under hot-dry conditions, was shown to be quite effectively in reducing low concentrations of hexavalent chromium in wastewater [44]. It was reported that Cr(VI) was reduced to Cr(III) without biosorption of either hexavalent or trivalent chromium; some extracellular activity of *P. chrysogenum* PTCC 5037 seems to be involved in this chromium bioreduction [44].

Another study described an equilibrium and modeling study on Cu(II) biosorption by live cells of *Penicillium cyclopium* using a fuzzy knowledge-based system [40]. Heavy metal uptake was found to be high and strongly dependent on pH, biomass and Cu ion concentrations in the solutions. The biosorption process was rapid, and in the first five minutes up to 75% of total Cu ions were deposited in the *P. cyclopium* surface [40].

Determination of the exact mechanism of complexation of an element is complicated, but can be achieved by X-ray absorption spectroscopy. This technique allows studies involving fast data collection, small samples, low concentrations, crystalline and amorphous solids and solutions. Additionally, X-ray absorption spectroscopy is a non-destructive, non-invasive method that could prove metal transformations at the ion-fungi interface and in its natural hydrated states. This technique was used to determine the speciation of toxic metals accumulated in *P. chrysogenum.* Experiments using cell walls of *P. chrysogenum* showed that carboxyl groups have greater affinity for Pb ions and preferentially bound lead at low metal concentrations and with an oxidation state of two. This oxidation state is more stable than four, and so there is a strong tendency for Pb(IV) compounds to react to give Pb(II) compounds, especially under acidic conditions [17].

## 3. Enzymes

Many filamentous fungi secrete high levels of plant cell wall hydrolyzing enzymes such as cellulases and xylanases into their culture media, and are employed for the hydrolysis of lignocellulosic materials [45]. In general, most of the ligninolytic fungi described require a high level of consumption of easily metabolized co-substrate to carry out lignin transformations. Studies with basidiomycetes have shown co-substrate consumption levels of up to 20 times (by weight) the amount of degraded lignin [46]. In a *P. chrysogenum* strain isolated from pine forest soils in Tenerife, lignin transformation with a low c-substrate requirement was observed [45]. This strain was able to transform about 83% and 90% of kraft and organosolv lignin after 30 days of incubation, respectively. Mineralization of kraft, organosolv, and synthetic dehydrogenative polymerisate lignins was also observed, but to a lesser extent than lignin transformation [45]. Nwodo-Chinedu *et al.* investigated the potential of *Penicillium chrysogenum* PCL501, isolated from wood-wastes, to produce extracellular proteins with cellulase activities [47]. This strain was capable of producing cellulase in basal media containing sawdust and cellulose [47]. Okafor *et al.* showed that the same fungus produced xylanase when cultured on media containing sawdust, oat-spelt xylan, wheat bran, and sugarcane pulp [48].

## 4. Polycyclic Aromatic Hydrocarbons

Until recently, we have not fully realized that when we are talking about polycyclic aromatic hydrocarbons (PAHs) “ubiquitous” literally means “everywhere”, and those vestige amounts of PAHs may be found in all surface samples of soil. From surface snow at the center of the Greenland icecap to urban areas all receive combustion-derived PAHs via the atmospheric circulation [49].

Polycyclic aromatic hydrocarbons (PAHs) are poorly soluble, hydrophobic organic compounds which are a class of environmental pollutants derived from the combustion of organic material, released into the environment by a variety of anthropogenic activities as well as natural geological processes. Therefore, PAHs represent an important class of contamination since the compounds are among the most frequently found in soil pollutants [49]. Their ubiquity along with their toxicity and mutagenic, makes them priority pollutants. The US Environmental Protection Agency (EPA) lists 16 PAHs as priority pollutants.

Many of the remediation technologies currently used for PAH-contaminated soil and water involve not only physical, chemical (photo- and chemical oxidation), but also biological methods. Biological transformations seem to be the prevailing route of their removal from sediments. The efficacy of this process as a removal mechanism depends on the type and concentration of the PAH present, soil characteristics, moisture content, temperature, microorganism, type and balance of nutrients, oxygen concentrations [50]. Concentration of oxygen gas in soil is generally thought to be one of the limiting factors in PAH degradation, not only because it is required as electron acceptor but also it serves as a co-substrate for oxygenase enzymes that initiate PAH metabolism [51,52]. Oxygen concentration varies according to soil depth and its availability depends on the amounts of substrate available and the type of soil. Additionally, in these processes hydrophobic solutes may react depending on the rate and extent of desorption from a solid surface or dissolution from a separate phase. This phenomenon is known as bioavailability and it is generally admitted to be one of the most important factors involved in the slow biodegradation of hydrophobic organic compounds in soil [53]. Therefore, oxygen and substrate availability affect the efficacy of aerobic polycyclic aromatic hydrocarbon remediation processes in contaminated soils.

The study with *Penicillium frequentans* showed that oxygen concentration has a significant effect on phenanthrene removal. *P. frequentans* was grown on sugarcane bagasse pith mixed with soil spiked supplemented with 200 mg/L of phenanthrene. A high removal rate was obtained for the higher oxygen concentrations, reaching 52% removal after 17 days with 20% oxygen. An opposite phenanthrene removal trend was found under low oxygen concentrations, reaching only 13% at close to 0% oxygen after 17 days of incubation. An explanation was presented based on adsorption of soil components due to the higher content in organic matter and clay [52].

Cyclodextrins are molecules that have the ability to solubilize pollutants, and consequently they are more accessible for the microorganisms. Cyclodextrins and its derivatives are cyclic oligomers of 1,4-α-D-linked glucose units [54] that have the capacity to desorb and complex PAHs.

Fluorene degradation by *P. italicum* in the presence of several cyclodextrins was reported [55]. Maltosyl-cyclodextrin, hydroxypropyl-β-cyclodextrin, partiallymethyl-β-cyclodextrin and β-cyclo-dextrin-sulfobutylether, all of them enhanced fluorene biodegradation. Maltosyl-cyclodextrin was shown to be the one that most increased fluorene degradation by *P. italicum* [55].

In general, fungi do not assimilate PAHs as the sole sources of carbon and energy, but require co-metabolites to detoxify them [56,57]. A substantial improvement in the rate of PAH degradation by *Penicillium janthinellum* VUO 10,201 was observed when glucose was added to basal salt medium. Under these conditions and in comparison to basal salt medium, PAH degradation rate by the VUO 10,201strain increased by 1.89- to 24.3-fold, and the lowest increase occurred for dibenz[*a,h*]anthracene, while the highest increase was observed for pyrene. *P. janthinellum* VUO 10,201 degraded substantial amounts of the four and five benzene ring PAHs in complex medium or basal salt-glucose medium. However, these PAHs did not support growth and were slowly degraded when supplied as a sole carbon source in basal salt medium [58].

The problem of low water solubility and soil adsorption that limit PAH availability to microorganisms could be solved by the use of surfactants. These molecules solubilize PAHs, increasing their concentrations in the aqueous phase and consequently should enhance their degradation. The influence of surfactants on solubilization and fungal degradation of fluorene was reported by Garon *et al.* [59]. It was found an enhancement of the degradation of fluorene by *P. chrysogenum* from 28 to 61% in the presence of Tween^®^ 80 (0.324 mM) after two days of incubation [59].

PAH degradation by filamentous fungi is mediated by either extracellular ligninolytic enzymes [60, 61], or by intracellular cytochrome P_450_ monooxygenases [60,62]. Metabolism of PAHs by *Penicillium* strains involves cytochrome P_450_ monooxygenase enzyme systems. The first steps of PAH oxidation result in the formation of monophenols, diphenols, dihydrodiols, and quinones [63]. In a second step, water-soluble conjugates such as sulfates and *O*-methyl conjugates, which are detoxification products, can be formed [64]. Anyway, both pathways yield PAH quinones as major oxidation products [63,65]. These metabolites have higher water solubility and reactivity than the parent PAH [66].

Pyrene, a four ring PAH, is known as not genotoxic and is commonly used as an indicator for monitoring PAH-contaminated wastes. However, its quinone-based metabolites are mutagenic and more toxic than the parent compound [67,68]. Therefore, it is important to know the pathways by which these products could be further degraded. Metabolism of pyrene by *Penicillium janthinellum* SFU403, a strain isolated from petroleum-contaminated soils, was described to proceed via hydroxylation to 1-pyrenol, followed by 1,6- and 1,8-pyrenequinones [63]. This strain was able to degrade only 20% of pyrene to water soluble products [63]. Later, these researchers reported that enhance in pyrene metabolism could be achieved by optimization of the carbon and nitrogen sources (glucose and nitrate concentrations) as well the bioconversion time. Pyrene hydroxylation was also detailed as occurred almost exclusively during the deceleration phase of culture growth [66]. In other strain of *P. janthinellum*, VUO 10,201, a similar effect on four and five-benzene-ring PAHs degradations were observed in basal medium and basal medium supplement with glucose. PAHs efficiency degradation by *P. janthinellum* VUO 10,201 increased in cultures supplemented with glucose [58].

Saraswathy and Hallberg described for the first time that fungi used pyrene as the sole carbon and energy source in liquid culture. Five pyrene-degrading strains of *Penicillium* were isolated from soil of a former gasworks site, and were identified as *P. simplicissimum*, *P. janthinellum*, *P. funiculosum, P. harzianum* and *P. terrestre*. Degradation of pyrene was directly correlated with biomass development. Comparison of dry weights and pyrene oxidation of *Penicillium* strains grown in both 50 and 100 mg/L pyrene ranked *P. terrestre* as the more efficient, followed by *P. simplicissimum, P. funiculosum, P. harzianum* and the strain with lower performance in pyrene degradation was *P. janthinellum* [68].

Another study with two *Penicillium ochrochloron* strains that were described as having an unique ability to metabolize pyrene as the sole carbon and energy source was reported [69]. One of the strains was able to degrade a maximum of 75% of 50 mg/L of pyrene during 28 days of incubation at 22 °C. The presence of pyrene influenced the size and shape of pellets as well as the density of mycelium and hyphal length of the *Penicillium* strains. The pellets with thick outer and dense inner surfaces showed higher degradation potential (about 89% of 50 mg/L of pyrene during 28 days of incubation) [69]. Table 2 shows the transformation of PAHs by several species of *Penicillium.*

The metabolism of pyrene by *Penicillium glabrum* TW 9424, a strain isolated from a site contaminated with PAHs, showed two novel metabolites in the fungal metabolism of polycyclic aromatic hydrocarbons (1-methoxypyrene and 1,6-dimethoxypyrene) [64].

Biological degradation is the major remediation process of PAH contaminated sites; however, its success has been limited by the failure to remove high-molecular-mass PAHs [49]. In contaminated soils, both bacteria and fungi are likely to be important in hydrocarbon degradation. Indeed, there may be potential for synergisms in hydrocarbon degradation between the two groups as each contain species that are specialized in different steps of hydrocarbon catabolism. Boonchan *et al.* found that a consortium of soil bacteria (*Stenotrophomonas maltophilia* VUN 10,010) and fungi (*P. janthinellum* VUO 10,201) was able not only to mineralize and grow on benzo[*a*]pyrene as the sole carbon and energy source, but also to achieve higher rates of its degradation [58]. The ubiquitous co-existence of bacteria and fungi in soil and their known catabolic cooperation, suggest that physical interactions between them may be of importance for PAH degradation.

## 5. Phenol and Its Derivatives

Phenol and its derivates are widely distributed as environmental pollutants due to their presence in the effluents of many industrial processes. Indeed, phenol is a major pollutant present in several industrial wastewaters such as petroleum refining, petrochemicals, basic organic chemical manufacture, pharmaceuticals, plastics manufacture, tannery, coal refining, pulp and paper manufacture, etc. [73]. Phenol is a troublesome contaminant causing skin irritation and it also contributes to off flavours in drinking and food processing waters. Due to its toxic nature the Environmental Protection Agency has set a water purification standard of less than 1 part per billion (ppb) of phenol in surface waters [74].

Halogenated compounds are important environmental pollutants of soil, water and air. In addition, the production and the utilization of fluorinated substances (pesticides, pharmaceutics, polyfluorinated polymers and tensides) have been increased enormously in the recent years [75]. Pentachlorophenol (PCP) is a biocide use in wood and timber preservation. The generalized application of PCP has severely contributed to contamination in wood-treatment plants. PCP is not only a persistent pollutant, but also toxic to all forms of life as it is an inhibitor of oxidative phosphorylation. Chlorophenols have been used for several purposes in the last 50 years such as wood preservation, domestic, agriculture and industry applications. The Preliminary Remediation Goals (PRGs) for the Superfund and Resource Conservation and Recovery Act (RCRA) programs for phenol, 2-chlorophenol, and pentachlorophenol are set to the limits of 37000 mg/kg, 63 mg/kg, and 3 mg/kg, respectively, for residential soils and 22000 μg/L, 30 μg/L, and 0.56 μg/L, respectively, for groundwater [76]. Wastewater containing phenols and phenolic compounds need careful treatment before discharge into the receiving bodies of water. They are currently removed by chemical or physical methods, including solvent extraction [77], membrane separation [78], activated carbon adsorption [79], sonication [80], Fenton oxidation [81], electrochemical oxidation [82], and ozonation [83]. In spite of the fact some of these methods have been shown to be effective in detoxification of phenols, most of them are not only expensive, but also lead to the formation of secondary toxic materials or lower mineralization or severe operating conditions, and are applicable to a limited phenol concentration range. Biological degradation has been utilized as an alternative, since it has low associated cost and leads to complete mineralization of the xenobiotic [84].

Fungi appear to be predominantly involved in metabolizing those xenobiotics of relative low solubility and high adsorptivity. Several *Penicillium* strains have the ability to transform them into products that are less mutagenic that the parent compound. In fact, the genus of *Penicillium* is a good hydrocarbon-assimilating strain. Scow *et al.* investigated in liquid medium the mineralization of phenol at low concentrations by *Penicillium* sp. isolated from silt loam that had been amended with 50 μg/g of phenol [85]. At concentrations ranges from 1.0 to 500.0 ng/mL, the rate constant of mineralization of phenol by this organism decreased with a lowering of the initial substrate concentration. Inoculum density affects not only the rate of mineralization as well the acclimation period: a lower the inoculum density implied lower maximum rate of mineralization and longer acclimation period [85]. Hofrichter *et al.* reported the utilization of phenol by *Penicillium frequentans* Bi 7/2 as the sole carbon and energy source. Phenol is degraded by the *ortho*-pathway with catechol as first intermediate product [86]. After 22 days of incubation *Penicillium simplicissimum* SK9117, effectively degraded 8.5 mM of phenol used as the sole source of carbon and energy. The catabolism of phenol produced catechol, hydroquinone, and *cis,cis*-muconic acid [87]. *S*tudies conducted with a halotolerant strain of *Penicillium chrysogenum* isolated from a salt mine (Algarve, Portugal) have shown that phenol could be complete degraded by this strain, and the products did not present toxicity. Phenol biodegradation was done with a concentration of 58.5 g/L of sodium chloride. *P. chrysogenum* CLONA2 strain was able to a rapid detoxification of 300 mg/L of phenol under batch conditions after 100 h of incubation [88]. Later, other study with the same fungus showed degradation of up to 300 mg/L of phenol and resorcinol in mineral salts medium with 58.5 g/L of sodium chloride. When phenol and resorcinol were together in low concentrations (< 15 mg/L and < 30 mg/L, respectively), phenol enhanced resorcinol degradation. *P. chrysogenum* CLONA2 metabolized phenol faster than resorcinol when present as a sole carbon source [89]. In spite of until now, only one pathway has reportedly been found for metabolism of phenol in the genus *Penicillium*, there is evidence that supports two possible routes, as it was described for *Aspergillus fumigatus* ATCC 28282 [87, 90]. In *P. simplicissimum* SK9117 and *P. chrysogenum* CLONA2, both catechol and hydroquinone were detected as metabolic products of phenol. In one route, phenol hydroxylates at the *ortho* position to form catechol, and in another route phenol hydroxylates at the *para*-position to produce hydroquinone (Leitão *et al.*, unpublished results).

Marr *et al.* described a *Penicillium* strain, *Penicillium simplicissimum* SK9117, able to grow on monofluorophenol. *P. simplicissimum* SK9117 was isolated from a sewage plant, and was able to metabolize 3-, 4-chlorophenol, and 4-bromophenol in the presence of phenol. The metabolism of all monofluorophenols is enhanced in the presence of phenol, resulting in the simultaneous disappearance of substrate (0.5 mM) and co-substrate (1 mM). When *P. simplicissimum* SK9117 used monofluorophenols as the sole carbon and energy source, growth was supported and fluoride ions released. The equimolar release of fluoride ions indicates complete mineralization of 3- and 4-fluorophenols.On the other hand, *P. simplicissimum* SK9117 was not able to utilize monobromo-, and monochlorophenols as carbon and energy source. 3-Chlorophenol was transformed by cometabolism to chlorohydroquinone, 4-chlorocatechol, 4-chloro-1,2,3-trihydroxybenzene, and 5-chloro-1,2,3-trihydroxybenzene. 4-chlorophenol was completely cometabolized, and only 4-chlorocatechol was observed as transient product. Degradations of chlorophenols were reported in other *Penicillium, P. frequentans* Bi 7/2. This fungus, when pregrown in phenol, metabolized monochlorophenol via the corresponding catechols and muconic acids [86]. Later, the cometabolic degradation of 2,6-dimethylphenol and o-cresol by *P. frequentans* was described [91]. Wunderwald *et al.* showed that the same fungus, using phenol as the only carbon and energy source, transformed difluorinated phenol into corresponding difluorocatechol [92].

A study with *Penicillium camemberti* has shown removals 86% of PCP and 53% of 2-chlorophenol in batch culture with Tween^®^ 80 after 21 days of incubation. In addition, under up-flow tubular column reactor conditions a removal of 77% pentachlorophenol from adsorbable organic halogen (concentration of 63.4 mg/L) was achieved after four days of incubation [94]. *Penicillium* degradation of phenols, chlorophenols and pentachlorophenol is listed in Table 3.

## 6. Wastewater and Waste

### 6.1. Olive Mill Wastewater

Olives grown in the Mediterranean countries constitute about 98% of the global production. Large quantities of olive mill wastewater (OMW) are produced during the manufacture of oil by traditional milling and press processes. This wastewater is a stable solution made up of vegetation, water from olives, washing and process water, olive pulp and oil. Borja *et al.* reported a release of 2.5 L of waste per liter of oil produced [95]. Conventional methods are not effective in the treatment of OMW. Electrolysis is one of the few methods with good results for the treatment of OMW [96]. Aerobic methods appear to be suitable, as one of the biggest problems of these wastewaters are phenolic compounds and their toxicity. Indeed, OMW contains phenolic, tannin and lignin compounds. *Penicillium* strains possess high catabolic enzyme activity and can utilize a wide variety of simple aromatic compounds. Therefore, these microorganisms exhibited a marked capacity for the detoxification of OMW, removing completely its antibacterial activity. Cultivation of OMW with *Penicillium* P4, one of the seven strains of *Penicillium* isolated from undiluted OMW that produce more biomass after 20 days of incubation, caused phenolic reduction of 54%, and COD reduction of 61%. Beside that the process resulted in a decolorization of 80% and no antibacterial activity against *Bacillus megaterium* was observed [97].

### 6.2. Vinasses

In Europe and USA a large number of industries produce ethanol by fermentation-destillation. These industries produce large quantities of high strength liquid wastes called vinasses, approximately 9–14 L of wastewater per liter of ethanol produced. The wastes generated by this kind of industry are strongly acidic (pH: 4–5) and have a high organic content (COD range of 50–100 g/L) [98]. Vinasses contain more than 10 different phenolic compounds and their polymers. These compounds are difficult to biodegrade, but usually they are treated by anaerobic digestion. Due to the presence of phenolic compounds the efficiency of anaerobic treatment could be improved by prior aerobic treatment. The pretreatment reduced not only the COD, phenolic concentration but also the toxicity of the system. Jimenez *et al.* reported the degradation of phenolic compound in vinasse by *Penicillium decumbens* in batch regime [99]. The initial concentration of substrate and phenolic compounds were 42.6 g COD/L and 0.21 g gallic acid/L, respectively. This fungus significantly decreased the total phenolic compounds in vinasses aerobically without the need for any nutrient supplements in the medium. A maximum value of phenols removal of 74% was achieved after three days’ treatment. *P. decumbens* produces a decolourization of the vinasses from the first day of incubation. Higher reductions in colour were achieved between the 4th and 5th days of treatment, with the best result being 41% of the initial colour removed after 100 hours of cultivation [98]. Later, the same research group carried out a comparative kinetic study on the anaerobic digestion of untreated and previously treated (with *Penicillium decumbens*) vinasses in continuous-flow stirred tank reactors [98]. The COD concentrations of reactors of untreated and treated vinasses were fed with 80.5 g/L and 23.0 g/L, respectively. The results obtained in the case of treated vinasses for kinetic constants, maximum specific growth rate and the model kinetic constant for methane production, were 9.6 and 6.9 times higher than those of untreated vinasses. The maximum volumetric methane production rate was 43% higher for pretreated vinasses than for untreated vinasses [98].

### 6.3. Coffee Residue

Coffee is one of the most important beverages of the world, and about one million tons of it is produced yearly in more than 50 countries. Brazil is the largest producer of coffee and hence of coffee residues in the world. The Brazilian government agency CONAB has released its first official estimate for the Brazilian coffee crop for 2009/10, together with the final official estimate for 2008/09. The average estimate for total production in 2009/10 is 37.8 million bags, with *Arabica* variety production forecast to reach 27.6 million bags and *Robusta* variety production estimated at 10.3 million bags [100]. At different stages, from harvesting to the processing and consumption, residues are generated formed by coffee husk or pulp (depending if it is dry or wet process), leaves and spent ground. Coffee residue is composed mainly of cellulose, galactomannan, pectin, caffeine, tannins and polyphenols. Although part of the residue is used for producing compost, the direct application of untreated coffee residues to soil is not possible as it inhibits the growth of several crops. Fujii and Takeshi have shown that from 25 strains isolated with coffee residue degrading activity, all of the fastest degraders were suggested to be *Penicillium* spp. [101] These strains have cellulolytic, mannolytic and pectinolytic activities. Considering that cellulose, pectin and galactomannan in coffee residue are degraded simultaneously by several species, it was suggested that *Penicillium* plays an important role in degradation of coffee residue [101]. Caffeine degradation by *Penicillium curstosum* in roast coffee infusions was reported when caffeine concentration were about 0.45–0.59 mg/mL [102]. Roussos *et al.* investigated the effect of additional inorganic and organic nitrogen sources in caffeine degradation by *Penicillium verrucosum*. In solid state fermentation of coffee pulp total degradation of caffeine was observed in the absence of any added nitrogen source after 72 h of fermentation, in spite of the limited growth of the culture. The presence of a additional source inhibiting caffeine degradation, it was proposed that *P. verrucosum* used caffeine as a nitrogen source [103]. In another study two *Penicillium* species strains were isolated from coffee pulp. These strains had the ability to degrade almost 100% of caffeine in liquid medium [104]. Silva *et al.* have found several *Penicillium* species at four stages of maturation and processing of coffee cherries of *Coffee arabica* in Brazil. A total of fourteen *Penicillium crustosum*, three *Penicillium restrictum*, two *Penicillium implicatum*, and one *Penicillium citrinum* strain were isolated. The potential of these strains were not studied, but the species identified include members all known to have pectinase and cellulase activities [105].

## 7. Conclusions

In this review it has been shown that *Penicillium* spp. plays an important role as natural environmental remediation agents in the ecosystem. Studies have shown the considerable potential of *Penicillium* strains as a naturally, abundant and cheap source for heavy-metal environmental reduction. In this process, fungi act as biosorbents. Their efficiency as adsorbents depends on the capacity, affinity and specificity including physico-chemical nature. Removal of heavy metals from aqueous solution could be achieved by using active, inactive and even dead biomass. The two last processes constitute an innovative and alternative technology for up-take of heavy metals. Biosorption offers an economically feasible technology for efficient removal and recovery of metal(s) from aqueous solution. This process could very interesting, once it could be applicable for treatment of dilute effluent that is originated either from a primary wastewater treatment or industrial plant. The major difficulties, at present, lies with the lack of knowledge on development of metal-resistant species and the performance of binary and ternary metal systems.

*Penicillium* strains, as soil fungi, showed ability to produce extracellular enzymes and metabolize hydrocarbons. The capacity of *Penicillium* strains to produce cellulose, mannanase and pectinase indicated that these strains should be able to use several agricultural wastes, and the potential for production of these costly enzymes should be considered for biotechnological applications. Some of these strains are not only able to oxidize, but also mineralize hydrocarbons, such as phenol, halogenated compounds and PAHs. Their efficiency depends on several factors such as co-substrate and inorganic macronutrient availability, mainly nitrogen and phosphorus. Recently, the use of fungal-bacterial co-cultures has demonstrated the detoxification of PAHs. Little is known about the biodegradation of complex mixtures of PAHs by fungi. Degradation of phenol and phenolic compounds by *Penicillium* strains has been achieved successfully at laboratory level despite limited work. However, further research is necessary for identification of metabolites and to elucidate the enzymatic pathways used in the biodegradation process. Genes encoding enzymes involving degradation of these pollutants must be cloned sequenced, and characterized, in order to open a new era in *Penicillium* applications for the detoxification of different xenobiotic compounds.

Industrial processes also use salts and frequently release brine-effluent into the environment. *Penicillium* species showed to be able to grow at high concentrations of salt as well as in its absence, while also processing high resistance to heavy metals and hydrocarbon degradation efficiency, could be used as agents for abatement of these pollutants in hypersaline conditions, as well as in non-saline environment.

A major concern expressed by several researchers in the bioremediation field is the lack of applicability of laboratory research to actual large-scale problems. Critical analysis reveals that there are relatively few reports of pilot and full-scale studies using *Penicillium* strains. Perhaps, the costs involved in projects, and the lack of control of natural environmental variables have delayed the implementation of fungi, and *Penicillium* strains in particular, in the bioremediation field. Therefore, so far we are only able to talk about the potential of *Penicillium* species in the field of bioremediation.

## Figures and Tables

**Figure 2.**
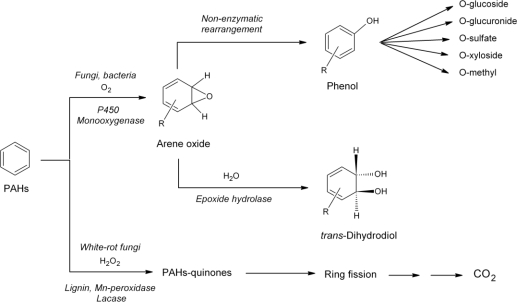
Initial steps in the degradation pathways of polycyclic aromatic hydrocarbons (PAHs) by fungi. Adapted from Cerniglia and Sutherland [60].

**Figure 3.**
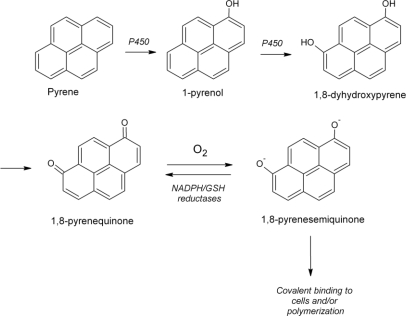
Pyrene metabolism by *P. janthinellum* SFU403. Adapted from Launen *et al.* [66].

**Figure 4.**
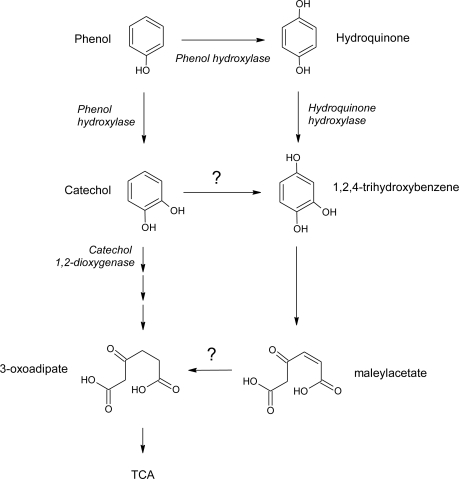
Possible pathway for the metabolism of phenol in *P. chrysogenum* CLONA2 (Leitão and Mendes, unpublished results).

**Table 1.** Heavy-metal adsorption capacities of several *Penicillium* strains. N/A: data not available.

**Biosorbent**	**Ion**	**Adsorption capacity (mg/g)**	**pH**	**Temperature (°C)**	**Concentration (mg/L)**	**Biomass (g/L)**	**Reference**
*P. simplicissimum*	Cd(II)	52.50	5.0	28	200.0	1.0	[31]
*P. chrysogenum*	Cd(II)	11.0	4–5	N/A	N/A	N/A	[18]
	Cd(II)	56.0	N/A	N/A	N/A	N/A	[36]
	Cd(II)	21.5	6.0	21	N/A	7.7	[37]
*P. canescens*	Cd(II)	102.7	5.0	N/A	N/A	N/A	[38]
*P. digitatum*	Zn(II)	9.70	5.5	25	25.0	7.0	[32]
*P. simplicissimum*	Zn(II)	65.60	5.0	28	250.0	1.0	[31]
*P. chrysogenum*	Zn(II)	6.5	4–5	N/A	N/A	N/A	[18]
	Zn(II)	13.0	6.0	21	N/A	7.7	[37]
*P. spinulosum*	Zn(II)	0.2	N/A	N/A	N/A	N/A	[39]
*P. simplicissimum*	Pb(II)	76.90	5.0	28	250.0	1.0	[31]
*P. chrysogenum*	Pb(II)	116.0	4–5	N/A	N/A	N/A	[18]
	Pb(II)	96.0	6.0	21	N/A	7.7	[37]
*P. canescens*	Pb(II)	213.2	5.0	N/A	N/A	N/A	[38]
*P. chrysogenum*	Cu(II)	11.7	6.0	21	N/A	7.7	[37]
*P. cyclopium*	Cu(II)	50.0	4.5	N/A	150.0	1.0	[40]
*P. spinulosum*	Cu(II)	0.4–2	N/A	N/A	N/A	N/A	[39]
*P. italicum*	Cu(II)	N/A	N/A	N/A	N/A	N/A	[33]
*P. canescens*	As(III)	26.4	5.0	N/A	N/A	N/A	[38]
	Hg(II)	54.8	5.0	N/A	N/A	N/A	[38]
*P. purpurogenum*	Cr(VI)	36.5	6.0	20	750.0	N/A	[35]
*P. chrysogenum*	U(VI)	70.0	4–5	23	N/A	N/A	[41]
	Th(IV)	142.0	4–5	23	N/A	N/A	[41]
*P. italicum*	Mn(II)	N/A	N/A	N/A	N/A	N/A	[33]
	Fe(III)	N/A	N/A	N/A	N/A	N/A	[33]
	Ni(II)	N/A	N/A	N/A	N/A	N/A	[33]
	Co(II)	N/A	N/A	N/A	N/A	N/A	[33]

**Table 2.** *Penicillium* sp. transformation of polycyclic aromatic hydrocarbons. N/A: data not available.

**Microorganism**	**PAHs**	**Depletion capacity (%)**	**Duration (days)**	**Concentration (mg/L)**	**Reference**
*P. canescens*	Fluorene	61	2	5.0	[55]
*P. janczewskii*	Fluorene	79	2	5.0	[55]
*P. montanense*	Fluorene	62	2	5.0	[55]
*P. simplicissimum*	Fluorene	72	2	5.0	[55]
*P. restrictum*	Fluorene	50	2	5.0	[55]
*P. chrysogenum*	Fluorene	28	2	5.0	[59]
*P. italicum*	Fluorene	28.4	2	5.0	[59]
*P. harzianum*	Pyrene	65.0	28	50.0	[68]
	Pyrene	33.7	28	100.0	[68]
*P. terrestre*	Pyrene	75.0	28	50.0	[68]
	Pyrene	67.0	28	100.0	[68]
*P. ochrochloron*	Pyrene	75.0	75	50.0	[69]
*P. chrysogenum*	Pyrene	18.9	2	10.0	[70]
*P. aurantiogriseum*	Pyrene	21.0	2	10.0	[70]
*P. crustosum*	Pyrene	0.2	2	10.0	[70]
*P. decumbens*	Pyrene	22.2	2	10.0	[70]
*P. griseofulvum*	Pyrene	16.5	2	10.0	[70]
*P. janczewskii*	Pyrene	17.9	2	10.0	[70]
*P. janthinellum*	Pyrene	24.3	2	10.0	[70]
*P. roqueforti*	Pyrene	16.5	2	10.0	[70]
*P. rugulosum*	Pyrene	9.2	2	10.0	[70]
*P. simplicissimum*	Pyrene	24.9	2	10.0	[70]
*P. velutinum*	Pyrene	22.5	2	10.0	[70]
*P. janthinellum*	Pyrene	57.0	28	50.0	[68]
	Pyrene	31.5	28	100.0	[68]
*P. janthinellum*	Pyrene	100.0	4	100.0	[63]
*P. janthinellum*	Pyrene	3.3	56	250.0	[58]
VUO 10,201					
	Chrysene	12.2	56	50.0	[58]
	Benz[*a*]antracene	9.1	56	50.0	[58]
	Dibenz[*a,h*]anthracene	12.9	56	50.0	[58]
	Benz[*a*]pyrene	16.9	56	50.0	[58]
*P. janthinellum*	Benz[*a*]pyrene	100.0	7	N/A	[63]
*P. janthinellum*	Benz[*a*]pyrene	61.0	56	50.0	[71]
*P. frequentans*	Phenanthrene	52.0	N/A	200.0	[52]
*Penicillium* sp. M1	Phenanthrene	100.0	60	50.0	[72]
	Fluoranthene	50.0	60	50.0	[72]

**Table 3.** *Penicillium* sp. degradation of phenols, chlorophenols and pentachlorophenol. N/A: not available data.

**Microorganism**	**Phenolic concentration**	**Degradation rate (%)**	**Duration (days or hours)**	**Concentration (mM or mg/L)**	**Reference**
*P. simplicissimum*	Phenol	100	N/A	8.5 mM	[87]
*P. chrysogenum*	Phenol	100	100 h	300 mg/L	[88]
*P. chrysogenum*	Resorcinol	100	120 h	200 mg/L	[89]
*P. frequentans* Bi 7/2	2-Chlorophenol	100	180 h	0.75 mM	[93]
*P. simplicissimum*	3-Chlorophenol	83	22 d	0.5 mM	[87]
*P. frequentans* Bi 7/2	3-Chlorophenol	100	37 h	1.0 mM	[93]
*P. simplicissimum*	4-Chlorophenol	100	8 d	0.5 mM	[87]
*P. frequentans* Bi 7/2	4-Chlorophenol	100	2 d	1.4 mM	[93]
*P. simplicissimum*	2-Fluorophenol	100	4 d	0.5 mM	[87]
*P. frequentans* Bi 7/2	2-Fluorophenol	100	55 h	2.0 mM	[93]
*P. simplicissimum*	3-Fluorophenol	100	13 d	0.5 mM	[87]
*P. frequentans* Bi 7/2	3-Fluorophenol	100	25 h	2.0 mM	[93]
*P. simplicissimum*	4-Fluorophenol	100	7 d	0.5 mM	[87]
*P. frequentans* Bi 7/2	4-Fluorophenol	100	18 h	2.0 mM	[93]
*P. simplicissimum*	4-Bromophenol	90	28 d	0.5 mM	[87]
*P. frequentans* Bi 7/2	3,4-Dichlorophenol	100	75 h	30 mg/L	[93]
*P. frequentans* Bi 7/2	2,4-Dichlorophenol	82	180 h	30 mg/L	[93]
